# The emergence of novel SARS-CoV-2 variant P.1 in Amazonas (Brazil) was temporally associated with a change in the age and sex profile of COVID-19 mortality: A population based ecological study

**DOI:** 10.1016/j.lana.2021.100021

**Published:** 2021-07-02

**Authors:** André Ricardo Ribas Freitas, Otto Albuquerque Beckedorff, Luciano Pamplona de Góes Cavalcanti, Andre M. Siqueira, Daniel Barros de Castro, Cristiano Fernandes da Costa, Daniele Rocha Queiróz Lemos, Eliana N.C. Barros

**Affiliations:** a Faculdade de Medicina São Leopoldo Mandic de Campinas, Campinas-SP, Brazil; b Programa de Pós-graduação em Saúde Coletiva da Universidade Federal do Ceará, Fortaleza-CE, Brazil; c Instituto Nacional de Infectologia Evandro Chagas iocruz, Brazil; d Fundação de Vigilância em Saúde do Amazonas, Brazil; e Faculdade de Medicina do Centro Universitário Christus, Fortaleza-CE, Brazil; f Centro de Farmacovigilância, Segurança Clínica e Gestao de Risco do Instituto Butantan, Brazil

**Keywords:** Covid-19, SARS-CoV-2, Severity, Case fatality rate, Epidemiology, VOC, Variants

## Abstract

**Background:**

Since the end of 2020, there has been a great deal of international concern about the variants of SARS-COV-2 B.1.1.7, identified in the United Kingdom; B.1.351 discovered in South Africa and P.1, originating from the Brazilian state of Amazonas. The three variants were associated with an increase in transmissibility and worsening of the epidemiological situation in the places where they expanded. The lineage B.1.1.7 was associated with the increase in case fatality rate in the United Kingdom. There are still no studies on the case fatality rate of the other two variants. The aim of this study was to analyze the mortality profile before and after the emergence of the P.1 strain in the Amazonas state.

**Methods:**

We analyzed data from the Influenza Epidemiological Surveillance Information System, SIVEP-Gripe (Sistema de Informação de Vigilância Epidemiológica da Gripe), comparing two distinct epidemiological periods: during the peak of the first wave, between April and May 2020, and in January 2021 (the second wave), the month in which the new variant came to predominate. We calculated mortality rates, overall case fatality rate and case fatality rate among hospitalized patients; all rates were calculated by age and gender and 95% confidence intervals (95% CI) were determined.

**Findings:**

We observed that in the second wave there were a higher incidence and an increase in the proportion of cases of COVID-19 in the younger age groups. There was also an increase in the proportion of women among Severe Acute Respiratory Infection (SARI) cases from 40% (2,709) in the first wave to 47% (2,898) in the second wave and in the proportion of deaths due to COVID-19 between the two periods varying from 34% (1,051) to 47% (1,724), respectively. In addition, the proportion of deaths among people between 20 and 59 years old has increased in both sexes. The case fatality rate among those hospitalized in the population between 20 and 39 years old during the second wave was 2.7 times the rate observed in the first wave (female rate ratio = 2.71; 95% CI: 1.9-3.9], p <0.0001; male rate ratio = 2.70, 95%CI:2.0-3.7), and in the general population the rate ratios were 1.15 (95% CI: 1.1-1.2) in females and 0.78 (95% CI: 0.7-0.8) in males].

**Interpretation:**

Based on this prompt analysis of the epidemiological scenario in the Amazonas state, the observed changes in the pattern of mortality due to COVID-19 between age groups and gender simultaneously with the emergence of the P.1 strain suggest changes in the pathogenicity and virulence profile of this new variant. Further studies are needed to better understanding of SARS-CoV-2 variants profile and their impact for the health population.

**Funding:**

There was no funding for this study.

**Research in Context**

**Evidence before this study** Previous studies reported a higher case fatality rate among patients with COVID-19 caused by Variant of Concern B.1.1.7 (VOC Alpha) when compared to patients who had become infected with the previous strains. This increase was homogeneous among the different age groups. There is very limited information available about the case fatality rate associated with other VOCs, such as B.1.351 (Beta) and P.1 (Gamma). This knowledge is very important to support the decisions of public managers and health professionals.

**Added value of this study** This study quantifies the differences between overall case fatality rate, and case fatality rate among hospitalized patients with covid-19 by age and sex before and after the emergence of the variant P.1 in the Amazonas state. The results show an increase in the proportion of severe hospitalized COVID-19 cases in the younger age groups and an increase in the proportion of deaths among people between 20 and 59 years old for both sexes. Although both sexes had higher severity and case fatality rate in the second wave, this increase was greater among women. There was an increase in the proportion of patients without pre-existing risk conditions among severe cases and deaths in the second wave compared to the first.

**Implications of all the available evidence** The P.1 variant has a significantly different virulence and epidemiological profile from the lineages previously established in the state of Amazonas during the first wave of the pandemic, namely B.1.1.28, B.1.1.29, B.1.1.33. This difference in outcomes and epidemiological profile presented by the P.1 variant may indicate that new variants can also lead to changes in the clinical and epidemiological profile of COVID-19. Monitoring these aspects will be essential to define global public health responses to new variants that may arise.

Alt-text: Unlabelled box

## 1 Introduction

Since the beginning of the COVID-19 pandemic, in March 2020, there has been a concern about the possibility of the emergence of new variants of SARS-CoV-2 with greater transmissibility or virulence. In late 2020 and early 2021 three new strains of SARS-CoV-2 were identified worldwide and considered as a Variant of Concern (VOC). The variant B.1.1.7 (also known as 20I / 501Y.V1 or VOC-202012/01) has been identified in the United Kingdom and carries the N501Y mutation that increases the virus's affinity for the ACE-2 receptor, which may explain its rapid expansion [1,2], associated with the worsening of the epidemiological situation in the United Kingdom, Portugal and other European countries between December 2020 and January 2021 [1]. This strain was considered more transmissible and more lethal than the dominant strains in the United Kingdom previously [2,3]. The variant B.1.351 (501Y.V2 or 20H / 501Y.V2) has been identified in South Africa, and carries three important mutations (K417N, E484K and N501Y), and seems to be more transmissible and less vulnerable to antibodies generated by a previous infection or vaccine [4]. However, studies on the clinical severity associated with this strain are still remaining.

The P.1 variant (20J / 501Y.V3 or VOC-202101/02) was first identified in four travelers, in Japan, who were returning from the state of Amazonas (North region of Brazil) on January 2, 2021 [5]. The P.1 strain has a large set of mutations, among which the K417T, E484K and N501Y stand out [6]. Immediately after the identification of this variant, public health authorities warned of the potential risk of faster dissemination or worsening of the clinical outcomes of the COVID-19 cases. The proportion of samples of patients with COVID-19 in Manaus (capital of the Amazonas state) with strains identified as P.1, that did not circulate until November 2020, increased to 52.2% (CI95% 40.5-63.8) in December, and to 85.4% (CI95% 72.5-93.1) in January 2021 [7]. In the same time, an abrupt increase in the number of hospital admissions by COVID-19 was observed in Manaus, which caused the collapse of the local health system [8]. Initial studies estimated that the P.1 strain can be between 1.4 and 2.2 times more transmissible than its precursors [9], and this may help explain the rapid worsening of the epidemiological situation in that location. Although there has been a large increase in the number of deaths due to COVID-19 in the Amazonas state since December 2020, it is not yet possible to say whether this was due exclusively to the increase in the total number of cases associated with the crisis in health services or if there was also a change in the pattern of severity of the disease due to the circulation of a new variant [9].

With the objective to describe and to identify possible changes in the mortality profile associated temporally to the emergence of the P1 strain in the state of Amazonas, we used public data of COVID-19 cases registered at the national epidemiological surveillance system. Two distinct epidemiological periods were considered in our analysis: the peak of the first wave, between April and May 2020, and January 2021 (the second wave), the month in which the new variant came to predominate.

## 2 Methods

### 2.1 Study design

We carried out a retrospective study of COVID-19 cases reported to the Ministry of Health through the Influenza Epidemiological Surveillance Information System, SIVEP-Gripe (Sistema de Informação de Vigilância Epidemiológica da Gripe). SIVEP-Gripe is composed by two distinct databases, one for registering mild forms of COVID-19, as influenza-like illness (ILI) syndrome treated on an outpatient basis, and the for registering cases of Severe Acute Respiratory Infections - SARI), including COVID-19 hospitalized cases. The notification of suspected and confirmed cases of COVID-19 is mandatory in Brazil, both in public and private health services [10].

We accessed information about demographic data, self-reported ethnicity, clinical data, comorbidities, hospitalization, admission to the ICU, ventilatory support and onset dates of the symptoms, hospital admission and outcome. The databases were made available by the Ministry of Health through a public website with anonymized data. The analysis period included the year 2020 until epidemiological week 5 of 2021, with data exported on March 1^st^ 2021.

We included all patients with Severe Acute Respiratory Infection (SARI) classified as confirmed for COVID-19 by laboratory, clinical, clinical-epidemiological or clinical-radiological criteria, according to Brazilian guidelines that follow the recommendations of the World Health Organization [10]. Population estimative for 2020 by gender and age group were obtained from the Brazilian Institute of Geography and Statistics (Instituto Brasileiro de Geografia e Estatística, IBGE).

### 2.2 Data analysis

We calculated all rates by age group and gender and 95% confidence intervals (95% CI) were determined. To calculate the incidence rate, we divide the total number of cases of ILI COVID-19 infection registered at SIVEP-Gripe by the population by sex and age group. We calculated the mortality and the case fatality rates and dividing the total number of deaths confirmed by COVID-19 reported in SIVEP-Gripe by total population and by confirmed COVID-19 cases. To calculate the case fatality rate among hospitalized patients with COVID-19, we divided the number deaths by COVID-19 among admitted patients by number of confirmed hospitalized COVID-19 cases (we excluded non-hospitalized cases and deaths from this calculation). We used median and interquartile ranges (IQR) to present the numerical variables and calculate the frequencies and proportions for the categorical variables.

We divided the analysis into three different time periods: the period including April and May 2020 was defined as the first wave, the period between June and November 2020 was defined as the inter-epidemic period, and the month of January 2021 was defined as the second wave. We did not include the month of December 2020 in the study because it was the period in which the old strains of SARS-CoV-2 were replaced by the P.1 lineage, which is the lineage of interest in this study [7]. We also did not include cases with the symptoms onset beginning in February 2021 considering that most of them had not yet reached the final outcome or their investigation closing until the time of the database extraction. The analyzes were carried out considering the date of the symptoms onset and for the calculation of the case fatality rates, only the cases whose outcome was concluded were considered. The impact of epidemic waves on mortality was assessed comparing the case fatality rates among hospitalized patients by calculating the ratio of epidemic (first and second wave) and non-epidemic periods and their respective confidence intervals. We used the Mantel-Haenszel method to adjust the rate ratio and evaluate the null hypothesis of homogeneity in the increase in case fatality rates in different age groups.

The data were analyzed using the software Stata 9.2. We followed the recommendations of the REporting of studies Conducted using Observational Routinely-collected health Data (RECORD), checklist is in the appendix. As this is open and anonymous data, this analysis did not require approval from any research ethics committee.

**Role of the funding agency:** There was no funding for this study.

## 3 Results

**Figure 1:**
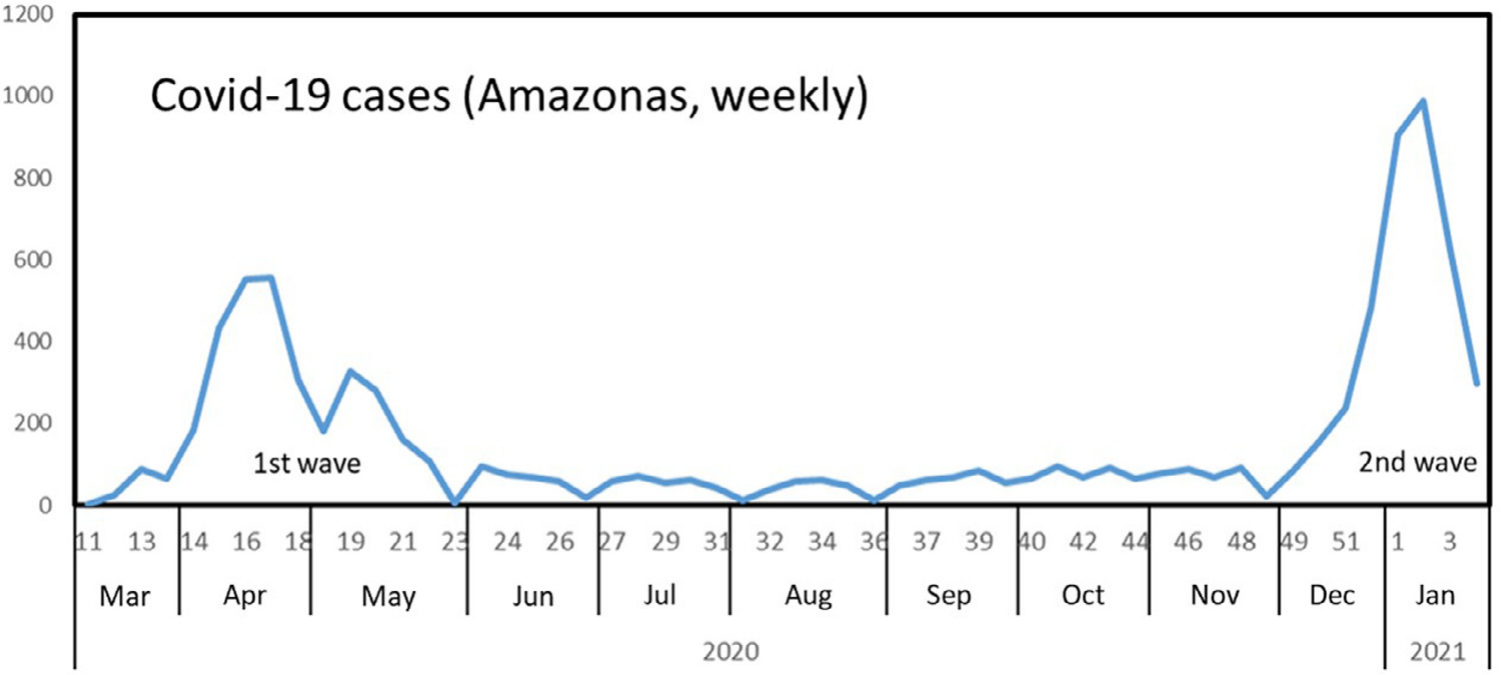
Weekly confirmed cases of COVID-19 in the State of Amazonas (Mar 2020- Jan 2021)

In the first wave of the COVID-19 pandemic in Amazonas state, 46,342 cases were recorded and, in the second wave, 61,273 cases, with the peak and slope of the curve also being greater in the second wave (figure 1). The second wave had a higher incidence and there was an increase in the proportion of cases of COVID-19 in the younger age groups (the distribution by sex and age group of ILI cases by COVID-19 are shown in Appendix 1).

The number of SARI cases in the first wave (April-May / 2020) was 6,816 and the number of deaths was 3,094. In the second wave (January / 2021), 6,142 SARI cases and 3,664 deaths were recorded (Table 1). The proportion of women among SARI cases increased from 40% (2,709) in the first wave to 47% (2,898) in the second, and the proportion of women among deaths from COVID-19 also increased from 34% (1,041) to 47 % (1,724) of the total deaths due to COVID-19 in the second wave. The median age among SARI cases was 59 years old (IQR = 45-71) in the first wave and 60 years old (IQR = 44-72) in the second wave. However, among those deceased by COVID-19 there was a great difference: in the first wave the median age was 69 years old (IQR = 60-79) and in the second one it was 65 years old (IQR = 54-76). Additionally, in the second wave, 56% (2,691) of SARI cases had no comorbidities, that represents a higher proportion when comparing with the 43% (3,919 cases) observed in the first wave. The proportion of patients without underlying conditions between SARI cases and COVID-19 deaths was higher in the first wave [43% (3,919 cases) and 31% (945 cases), respectively] than in the second wave [56% (2,691 cases) and 50% (1,842 deaths) respectively] (Table 1).

**Table 1:** Demographic, clinical data and evolution of patients with severe acute respiratory infection (SARI) confirmed in the first and second waves of COVID-19 in the State of Amazonas.

	Wave 1	Wave 2
	(April 1, to May 31, 2020)	(Jan 1, to Jan 31, 2021)
**SARI cases (n)**	6,816	6,142
**Deaths (n)**	3,094	3,664
**Sex, female**		
Among all SARI cases	2,709(40%)	2,898(47%)
Among deaths	1,051(34%)	1,724(47%)
**Age, yearsYears old**		
Among all SARI cases	60(44-72)	59(45-71)
Among deaths	69(60-79)	65(54-76)
**Residence area**		
Manaus city	4,339(64%)	4,238(69%)
Urban area	6,164(90%)	5,215(85%)
**Ethnicity**		
Pardo (mixed ethnicity)	5,329(78%)	5,123(83%)
White	483(7%)	472(8%)
Indigenous	208(3%)	72(1%)
Black	113(2%)	70(1%)
Asian	49(1%)	28(0%)
N.D.	634(9%)	377(6%)
**Symptoms**		
Fever	5,832(86%)	4,555(74%)
Cough	5,719(84%)	4,571(74%)
Sore throat	3,076(45%)	2,370(39%)
Dyspnoea	5,393(79%)	4,813(78%)
Diarrhea	1,182(17%)	863(14%)
Vomiting	637(9%)	483(8%)
Others	1,901(28%)	1,294(21%)
**Underlying conditions**		
Cardiopathy	1,900(28%)	1,258(20%)
Diabetes	1,688(25%)	1,077(18%)
Obesity	190(3%)	329(5%)
Neurological disease	160(2%)	90(1%)
Asthma	147(2%)	88(1%)
Chronic liver disease	64(1%)	29(0%)
Hematological disease	62(1%)	33(1%)
Other	1,849(27%)	1,015(17%)
**Without underlying conditions**		
Among all SARI	3,919(43%)	2,691(56%)
Among deaths	945(31%)	1,842(50%)
**Case management**		
Hospilatization	6,411(94%)	5,281(86%)
UTI	1,375(20%)	1,083(18%)
Invasive ventilation	1,251(23%)	1,126(25%)
Non invasive respiratory support	2,988(54%)	2,420(55%)
**Time (means, days) since symptoms onset until**		
Hospitalization	7(4-11)	8(4-11)
Death	17(12-28)	15(9.25-21)
Discharge	12(7-19)	15(10-22)
**Diagnostic criteria**		
Laboratory	6,258(92%)	4,259(69%)
Clinical-epidemiological	217(3%)	321(5%)
Clinical	59(1%)	686(11%)
Clinical-radiological	232(3%)	702(11%)
N. D.	50(1%)	174(3%)

There was an increase in the proportion of deaths between 20 and 59 years of age in both sexes. Among the female population, the proportion of deaths in this age group, in the first wave, was 24% (255 deaths) and increased to 33% (570 deaths) in the second wave. In the male population, the proportion of deaths between 20 and 59 years old was 24% (493 deaths) in the first wave and increased to 38% in the second wave (747 deaths) (Table 2).

**Table 2:** Confirmed deaths, mortality rate and case fatality rate by COVID-19 in the first and second waves in Amazonas state (2020-2021).

Sex, age		Deaths (all)				Mortality rate (deaths per 100.000pop)			Case fatality rate (deaths per 100 reported cases, appendix)				
	Population (X1000)	Apr-Mai 2020		Jan 2021		Apr-Mai 2020(a)	Jan 2021(b)	Rate Ratio (b/a)	Apr-Mai 2020(a)	Jan 2021(b)	Rate Ratio (b/a)		
Female		n	%	n	%			RR (95%CI)			RR (95%CI)	p	Mantel-Haenszel (p)
**0-19 ys**	789,306	8	0.8	11	0.6	1.0	1.4	1.38 (0.5-3.9)	0.5	0.3	0.69 (0.3-2)	0.4352	<0.0001
**20 - 39 ys**	729,310	57	5.4	112	6.5	7.8	15.4	1.96 (1.4-2.8)	0.6	0.8	1.36 (1-1.9)	0.0553	
**40 - 59 ys**	421,747	198	18.8	458	26.6	46.9	108.6	2.31 (2-2.7)	2.2	3.7	1.67 (1.4-2)	<0.0001	
**60 - 79 ys**	141,988	527	50.1	797	46.2	371.2	561.3	1.51 (1.4-1.7)	17.5	19.9	1.14 (1-1.3)	<0.05	
**80 and more**	19,738	261	24.8	346	20.1	1322.3	1753.0	1.33 (1.1-1.6)	38.8	60.2	1.55 (1.3-1.8)	<0.0001	
**All ages (crude)**	2,102,089	1,051	100.0	1,724	100.0	50.0	82.0	1.64 (1.5-1.8)	4.4	5.1	1.15 (1.1-1.2)	0.0004	
**All ages (M-H combined)**											1.34 (1.25-1.43)		
**% of female**	0	34%		47%									

**Male**													

**0-19 ys**	826,309	11	0.5	13	0.7	1.3	1.6	1.18 (0.5-2.9)	0.8	0.5	0.61 (0.3-1.5)	0.2358	<0.0001
**20 - 39 ys**	737,193	70	3.4	147	7.8	9.5	19.9	2.1 (1.6-2.8)	0.8	1.4	1.66 (1.2-2.2)	<0.001	
**40 - 59 ys**	426,202	423	20.7	600	29.8	99.2	140.8	1.42 (1.3-1.6)	5.0	6.0	1.2 (1.1-1.4)	<0.005	
**60 - 79 ys**	134,826	1086	53.2	895	46.3	805.5	663.8	0.82 (0.8-0.9)	30.1	24.7	0.82 (0.8-0.9)	<0.0001	
**80 and more**	13,591	453	22.1	285	15.4	3333.1	2097.0	0.63 (0.5-0.7)	61.8	60.8	0.98 (0.8-1.1)	0.8257	
**All ages (crude)**	2,138,121	2,043	100.0	1,940	100.0	95.6	90.7	0.95 (0.9-1)	9.1	7.1	0.78 (0.7-0.8)	<0.0001	
**All ages (M-H combined)**											0.97 (0.92-1.02)		
**% of male**	50%	66%		53%									
**Total**	4,240,210	3,094		3,664									

Mortality and case fatality rates increased with age in both sexes, in both waves. The overall female mortality rate was higher in the second wave than in the first, mortality rates were higher in all age groups. The differences in mortality rates between the waves were greater in the age groups between 20 and 39 years (rate ratio = 1.96 (95% CI: 1.4-2.8)) and 40 and 59 years (rate ratio = 2.31 (95% CI: 2-2.7)). The case fatality rate increased significantly in all female age groups from the age of 40, but the increase was greater between 40 and 59 years old (table 2).

The male mortality rate was lower in the second wave than in the first. However, the mortality and case fatality rates in the age groups between 20 and 59 years were higher in the second wave. Differently from the female population, the mortality and case fatality rates in the age groups of 60 to 79 years old and above 80 years of age were lower in the second wave than in the first one among males. There was no statistically significant difference in the case fatality rate among men over 80 years of age when comparing the two waves.

The case fatality rates among hospitalized patients were higher in the older ages in both sexes and in both waves (table 3). Comparing the first and second waves, we observed that among women there was an increase in the hospital mortality rate in all age groups. The increase in case fatality rates among female hospitalized patients was not homogeneous in the different age groups, the variation was greater in the age groups of 20 to 39 years (rate ratio = 2.71 (95% CI: 1.9-3.9), p = 0.0001) and 40 to 59 years (rate ratio = 2.1 (95% CI: 1.8-2.5), p <0.0001) and was not statistically significant among people under 20 years of age (table 3). Among men, there was an increase in the case fatality rate among hospitalized patients in all age groups, with the exception of those over 80 years of age. The increase in the hospital case fatality rate among men was greater in the age groups of 20 to 39 years (rate ratio = 2.7 (95% CI: 2-3.7), p <0.0001) and 40 to 59 years (rate ratio = 1.58 (95% CI: 1.4- 1.8), p <0.0001) (table 3).

**Table 3:** Hospitalizations, deaths, mortality rate and case fatality rate among hospitalized patients with COVID-19 in the first and second waves in Amazonas state (2020-2021).

Sex, age	Hospitalizations				In-hospital mortality				Case fatality rates for patients admitted				
	Apr-May 2020		Jan 2021		Apr-May 2020		Jan 2021		Apr-May 2020(a)	Jan 2021(b)	Rate Ratio (b/a)		
Female	n	%	n	%	n	%	n	%	deaths/100 admitted cases (95%CI)	deaths/100 admitted cases (95%CI)	RR (95%CI)	p	Mantel-Haenszel (p)
**0-19 ys**	114	4.5	54	2.2	8	0.8	9	0.6	7(2.2-11.9)	16.7(5.8-27.6)	2.38(0.8-7.1)	0.0821	<0.0001
**20 - 39 ys**	496	19.4	339	13.8	53	5.6	98	6.6	10.7(7.8-13.6)	28.9(23.2-34.6)	2.71(1.9-3.9)	<0.0001	
**40 - 59 ys**	693	27.2	765	31.2	178	18.7	412	27.6	25.7(21.9-29.5)	53.9(48.7-59.1)	2.1(1.8-2.5)	<0.0001	
**60 - 79 ys**	915	35.9	993	40.4	488	51.2	718	48.1	53.3(48.6-58.1)	72.3(67-77.6)	1.36(1.2-1.5)	<0.0001	
**80 and more**	333	13.1	304	12.4	227	23.8	257	17.2	68.2(59.3-77)	84.5(74.2-94.9)	1.24(1-1.5)	<0.05	
**All ages (crude)**	2551	100.0	2455	100.0	954	100.0	1494	100.0	37.4(35-39.8)	60.9(57.8-63.9)	1.63(1.5-1.8)	<0.0001	
**All ages (M-H combined)**											1.54(1.46-1.63)		
**% of female**	40%		46%		34%		47%						

**Male**													

**0-19 ys**	128	3.3	52	1.8	11	0.6	12	0.7	8.6(3.5-13.7)	23.1(10-36.1)	2.69(1.1-6.7)	<0.05	<0.0001
**20 - 39 ys**	495	12.8	355	12.6	66	3.5	128	7.6	13.3(10.1-16.6)	36.1(29.8-42.3)	2.70(2-3.7)	<0.0001	
**40 - 59 ys**	1197	31.0	1057	37.4	386	20.7	539	31.9	32.2(29-35.5)	51(46.7-55.3)	1.58(1.4-1.8)	<0.0001	
**60 - 79 ys**	1553	40.2	1093	38.7	1007	54.0	793	47.0	64.8(60.8-68.8)	72.6(67.5-77.6)	1.12(1-1.2)	<0.05	
**80 and more**	487	12.6	269	9.5	396	21.2	216	12.8	81.3(73.3-89.3)	80.3(69.6-91)	0.99(0.8-1.2)	0.885	
**All ages**	3860	100.0	2826	100.0	1866	100.0	1688	100.0	48.3(46.1-50.5)	59.7(56.9-62.6)	1.24(1.2-1.3)	<0.0001	
**All ages (M-H combined)**											1.27(1.22-1.32)		
**% of male**	60%		54%		66%		53%						
**Total**	6411		5281		2820		3182						

**Figure 2:**
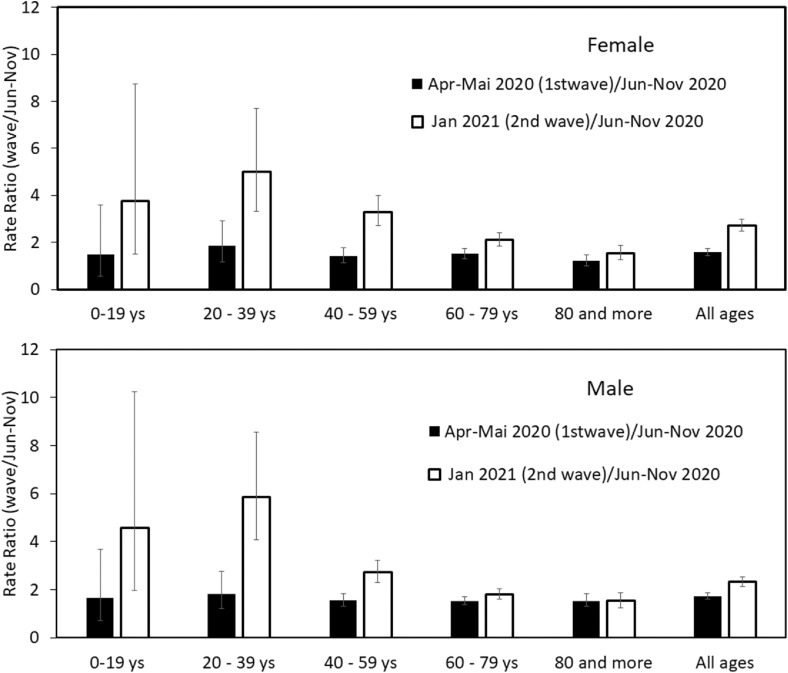
Case fatality rate ratio of patients hospitalized for COVID-19 in the State of Amazonas between epidemic and interepidemic periods (2020-2021), according to sex and age group. Black columns refer to the case fatality rate among hospitalized cases in the first wave / interepidemic period. White columns refer to the case fatality rate among hospitalized cases in in the second wave / inter-epidemic period. The error bar is the 95% confidence interval.

When comparing the hospital case fatality rates of the first and second waves with the inter-epidemic period (figure 2), we found that the first pandemic wave had higher hospital mortality rates than in the interepidemic period in both sexes (female rate ratio = 1.73 (95% CI: 1.6-1.9), p <0.0001; female rate ratio = 1.58 (95% CI: 1.4-1.7), p <0.0001) and in all age groups homogeneously. In the second wave there was a greater increase in female mortality (rate ratio = 2.58 (95% CI: 2.4-2.8), p <0.0001) than in male (rate ratio = 2.14 (95% CI: 2.0-2.3), p <0.0001), in addition to the growth, it was not homogeneous in the different age groups, showing a more significant increase in mortality in the age groups up to 59 years in both sexes.

## 4 Discussion

The analysis of mortality data suggests that there was a change in the mortality profile by sex and age group when comparing the first wave of the COVID-19 pandemic in Amazonas, in which strains B.1.1.28, B.1.1.29 predominated and B.1.1.33 of SARS-CoV-2, and the second pandemic wave, in which the emerging P.1 strain predominated. There was a higher proportion of women and the population between 20 and 59 years old of both sexes among deaths in the period in which the P.1 lineage predominated when comparing periods in which the previous lineages predominated. The global female mortality rate due to COVID-19 in the second wave was 1.64 times the mortality rate observed in the first wave (95% CI:1.5-1.8), but the change was greater in the age groups between 20 and 39 years and 40 to 59 years (ratio of rates, respectively = 1.96 (95%CI: 1.4-2.8) and 2.31 (95% CI: 2-2.7)), suggesting that these age groups were disproportionately more affected in the second wave than in the first. The overall mortality due to COVID-19 in males was similar when comparing the two waves, with a ratio of 0.95 (95% CI: 0.9-1.0). However, the findings suggest that the risk of death among men aged 20 and 39 in the second wave was more than double when compared to the first wave (rate ratio = 2.1 (95% CI: 1.6-2.8), p <0.0001). Among men aged 40 to 59 years the rate ratio was 1.42 (95% CI:1.3-1.6, p <0.0001) representing an increased risk in this age group as well. Conversely, among men aged 60 to 79 years and older than 80 years, mortality rates due to COVID-19 were lower in the second wave than in the first one (rate ratio = 0.82 (95% CI: 0.8-0.9) and 0.63 (95% CI: 0.5-0.7), respectively).

The case fatality rate among those hospitalized cases between 20 and 39 years old during the second wave was 2.7 times the rate observed in the first wave (female rate ratio = 2.71 (95% CI: 1.9-3.9), p <0.0001; male rate ratio = 2.70 (95% CI: 2.0-3.7), p <0.0001). It is known that, in addition to the severity of hospitalized cases, the hospital mortality rate varies according to the demand for health care and the availability of human and material resources [11,12]. However, the change in the age pattern was consistent in different mortality indicators (mortality rate, case fatality rate and case fatality rate among hospitalized patients), suggesting greater severity among young adults of both sexes and the general female population. These findings are also compatible with studies that showed that the viral load in P.1 infections was significantly higher than in non-P.1 infections in men between 18 and 59 years old (P = 0.0005), women between 18 and 59 years old (P <0.0001) and women over 59 years old (P = 0.0149); but not significantly different in men over 59 years old (P = 0.4624) [13]. This set of findings suggests a change in the virulence profile of SARS-CoV-2 coinciding with the emergence of the P.1 strain in the State of Amazonas. Reinforcing the hypothesis of a change in the pattern of virulence; there was a decrease in the median age among the deaths and an increase in the proportion of patients without pre-existing diseases among the cases of SARI and deaths due to COVID-19 (SARI) in the second wave (Table 1).

Mortality due to COVID-19 in Amazonas was greatly influenced by the overload of the local health system, which was collapsed in both the first and second waves, and a comparison between the epidemics and interepidemic periods were performed [11]. The proportion of SARI patients who were hospitalized in a single month second wave (86%, 5,281 hospitalizations) was lower than in the two months analyzed of the first wave (94%, 6,411 hospitalizations), suggesting that there was a greater shortage of beds in the hospital system during the second wave, which had to assist a larger demand from patients in a shorter period of time. These aspects could be associated with an increase in mortality rates, case fatality rate and hospital mortality as already observed in other situations [11,12]. The increase in the hospital mortality rate in the second wave could have been a strict consequence of overload in the health system that was more required than in the first wave, however, if this was the only explanation, the increase would be more homogeneous among the different age groups and sex as occurred in the first wave when compared to the inter-epidemic period (figure 2). According to the resolution of the Federal Council of Medicine (CFM No. 2,156 / 2016), in a situation in which there is a shortage of places for the number of patients, hospitalization places must be directed with priority to “patients who need interventions to support health care with a high probability of recovery and without any limitation of therapeutic support”. Thus, if scarcity was the justification for heterogeneity, what would be expected to see is a greater increase in mortality among the elderly and male patients, who are known to have a worse prognosis for this disease [11,12]. As observed in our results, when comparing the case fatality rate among admitted patients of the first wave with that one during the interepidemic period (period in which the lines B.1.1.28, B.1.1.29 and B.1.1.33 predominated), there was an overall increase in this rate (rate ratio> 1) and it was reasonably homogeneous between sexes and among different age groups. However, when we compared the second pandemic wave (which predominated the P.1 strain) with the interepidemic period, the difference in hospital case fatality rate was greater in the global female and among those under 60 years old in both sexes.

Considering the other VOC, the emergence of the B.1.1.7 strain in the United Kingdom was associated with a rapid worsening of the epidemiological picture with an increase in the estimated transmission rate of between 43–90% [2], at the same time there was an increase in the proportion of cases among young adults [1]. Analysis of a population-based cohort conducted by Public Health England found a higher risk of death for individuals infected with the B.1.1.7 strain of 1.65 (95% CI 1.21-2.25) when compared to the previous strains, however, similar evaluation have not been reported by age group [14]. The increase in the mortality rate among those infected by this new strain does not seem to have been a consequence of an increase in the hospital mortality rate, according to the results of a study based on inpatients carried out by the Coronavirus Clinical Information Network (COCIN) initiative in the United Kingdom [14]. ​​The emergence of the 501Y.V2 strain was associated with a rapid deterioration of the epidemiological situation and the transmissibility was estimated at 1.56 (95% credible interval l (CrI): 1.50-1.74) in relation to the precursors [15], this same study suggests that there may have been some increase in the severity of the disease, without further details.

In conclusion, our findings suggest that simultaneously with the emergence of P.1 lineage in the state of Amazonas, there was an increase in the proportion of deaths due to COVID-19 in the group of women and in the proportion of these deaths among populations between 20 and 59 years of both sexes. Also, there were relative increase in mortality rates, case fatality rate and hospital case fatality rate in the different age groups and sexes. Up to our knowledge, this is the first evidence that P.1 (and maybe other VOCs) can affect men and women of different age groups differently when compared to previous strains, suggesting changes in the profiles of pathogenicity and virulence. New studies are still needed to be carried out integrating areas of epidemiology, virology, clinic and immunology to expand knowledge about the dynamics of COVID-19 in the context of the VOCs circulation to support the recommendations for prevention and control measures.

### 4.1 Study limitation

This ecological study has some limitations inherent to the study design, as following: the use of secondary data from health information systems in Brazil when in one hand represents a wide population coverage and have been widely used in epidemiological analyzes of COVID-19 pandemic in Brazil [11], it may present problems in the quality and completeness of the recorded data; this is an initial analysis in which a limited range of available variables were evaluated, and additional individual risk factors were not deeply explored. Despite these limitations, the results presented in this study may suggest a potential causal association between exposure to the new SARS-CoV-2 variant (P.1) and the change in epidemiological profile in Brazil that should be considered as hypotheses to be evaluated in subsequent studies.

## Contributors

ARRF: conceptualization, data curation, formal analysis, investigation, methodology, project administration, validation, visualization, writing – original draft, and writing – review & editing. OAB have accessed verified the underlying data, writing – review & editing. LPGC: writing – review & editing. AMS: writing – review & editing. DBC: data curation, have accessed verified the underlying data. CFC: data curation, have accessed verified the underlying data. DRQL: writing – original draft, writing – review & editing. ENCB: writing – original draft, writing – review & editing

## Declaration of interests

All authors declare no competing interests.

## Appendix Supplementary materials

Image, application 1
